# Clinical Prognostic Value of RNA Viral Load and CD4 Cell Counts during Untreated HIV-1 Infection—A Quantitative Review

**DOI:** 10.1371/journal.pone.0005950

**Published:** 2009-06-17

**Authors:** Eline L. Korenromp, Brian G. Williams, George P. Schmid, Christopher Dye

**Affiliations:** 1 Department of Public Health, Erasmus MC, University Medical Centre Rotterdam, Rotterdam, The Netherlands; 2 The Global Fund to Fight AIDS, Tuberculosis and Malaria, Geneva, Switzerland; 3 The South African DST/NRF Centre for Epidemiological Modelling and Analysis (SACEMA), University of Stellenbosch, Stellenbosch, South Africa; 4 HIV/AIDS Department, World Health Organization, Geneva, Switzerland; 5 Office of HIV/AIDS, Tuberculosis, Malaria and Neglected Tropical Diseases, World Health Organization, Geneva, Switzerland; McGill University Health Center, Montreal Chest Institute, Canada

## Abstract

**Background:**

The prognostic value of CD4 counts and RNA viral load for identifying treatment need in HIV-infected individuals depends on (a) variation within and among individuals, and (b) relative risks of clinical progression per unit CD4 or RNA difference.

**Methodology/Principal Findings:**

We reviewed these measurements across (a) 30 studies, and (b) 16 cohorts of untreated seropositive adults. Median within-population interquartile ranges were 74,000 copies/mL for RNA with no significant change during the course of infection; and 330 cells/µL for CD4, with a slight proportional increase over infection. Applying measurement and physiological fluctuations observed on chronically infected patients, we estimate that 45% of population-level variation in RNA, and 25% of variation in CD4, were due to within-patient fluctuations. Comparing a patient with RNA at upper 75^th^ centile with a patient at median RNA, 5-year relative risks were 1.4 (95% CI 1.2–1.7) for AIDS and 1.5 (1.3–1.9) for death, without change over the course of infection. In contrast, for a patient with CD4 count at the lower 75^th^ centile, relative risks increased from 1.0 at seroconversion to maxima of 6.3 (4.4–8.9) for AIDS and 5.5 (2.7–10.1) for death by year 6, when the population median had fallen to 300 cells/µL. Below 300 cells/µL, prognostic power did not increase, due to a narrower CD4 range.

**Conclusions:**

Findings support the current WHO recommendation (used with clinical criteria) to start antiretroviral treatment in low-income settings at CD4 thresholds of 200–350 cells/µL, without pre-treatment RNA monitoring – while not precluding earlier treatment based on clinical, socio-demographic or public health criteria.

## Introduction

CD4 cell counts (CD4) and RNA viral loads (RNA) are the two most commonly used prognostic markers of the clinical progression of HIV infection [1]–[3]. In the chronic stage of infection, the level of viral replication, as measured by the concentration of RNA in the blood, largely determines the time from initial infection to AIDS and death: high initial RNA is a marker for rapid progression, and in all individuals clinical progression is preceded by an increase in RNA [4]–[8]. As infection progresses, CD4 declines [9], [10] and should, in principle, provide a measure of the remaining time to AIDS and death. Despite a broad consensus on these prognostic patterns, recent observations of poor correlation between RNA and CD4 decline rates in adult patients have led to a renewed debate over the relative merits of RNA and CD4 as prognostic indicators in the absence [11], [12] and presence [13] of highly active antiretroviral treatment (ART).

As ART becomes increasingly available in low-income countries [14], knowledge of the natural history of HIV infection and the prognostic value of RNA and CD4 measurements is increasingly important:

- On a population basis, to help determine at what threshold it is most effective, and cost-effective, to begin therapy of treatment-naïve patients [15];
- On an individual basis, to refine management of treatment-naïve patients [15];
- To optimize treatment allocation among patients in settings where available resources cannot (yet) meet the full demand for ART [16];
- To improve the empirical basis for prognostic and epidemiological models of the impact and cost-effectiveness of ART [16].

The prognostic value depends on (i) the relative risk of progression per unit difference in RNA or CD4, and (ii) the extent to which RNA and CD4 levels vary within populations. The interpretation of published data on these two components of prognostic value is complicated by several factors. First, prognostic values are likely to vary as the infection progresses. Unfortunately, in many studies dates of seroconversion are not known or known only with considerable uncertainty. Further, among (and within) studies with known dates of seroconversion, the timing of RNA or CD4 ‘baseline’ measurements varies, as does the subsequent duration of follow-up. Second, studies use a variety of outcome measures and analytical methods to interpret observed associations.

To determine the comparative prognostic values of RNA and CD4 over the course of untreated infection, we review and analyze quantitative data on progression risks associated with high RNA and low CD4, and within-population variability in both markers, from high-quality cohort studies of HIV-seropositive adults. All observations are ‘standardized’ in types of outcome measure, and in time relative to the intervals since seroconversion of patients. As a complementary approach, magnitudes of within-patient (laboratory and physiological) and among-patient variation are reviewed and compared between RNA and CD4. Results are discussed in the light of treatment initiation recommendations and CD4/RNA testing algorithms that are either in use or have been proposed for low-income settings.

## Methods

### Literature review

Studies on RNA, CD4 and their variation within HIV-1 infected populations, and the risks of AIDS and death associated with high RNA or low CD4 published up to May 2008 were identified by PubMed search. Search terms included (HIV-1 OR AIDS), (RNA OR viral load), CD4, (longitudinal OR cohort OR seroconver* OR prognos*), and (AIDS OR mortality OR survival OR progress* OR (natural history)). Relevant publications (in English, French or Spanish) were traced backward and forward in time to identify additional data on the same patient cohort. In instances needed, authors were contacted to obtain or verify data from eligible studies. Study selection and analyses adhered to the *MOOSE Guidelines for Meta-Analyses and Systematic Reviews of Observational Studies* (see Text S1) [17].

Outcome measures considered were:

- Relative risks associated with high RNA or low CD4 for subsequent development of clinical AIDS and/or death.
- RNA and CD4 median or mean levels, with interquartile ranges (IQRs) or other centiles (e.g. 10^th^ and 90^th^), minimum, maximum and standard deviations (SD).

Studies were included if:

- They reported one or more relevant outcome measures for HIV-1 seropositive adults aged≥15 years. All types of risk population, including men having sex with men, injection drug users, haemophiliacs, and heterosexually infected men and women, were eligible, as well as all types of study samples including general population surveys, clinic or hospital patient reports, sero-incident cohorts and sero-prevalent surveys. However, study groups were not allowed to have been selected according to their subsequent prognosis or the presence or absence of symptoms during primary infection.
- They reported the average duration of infection in the study population at time of the relevant ‘baseline’ RNA or CD4 measurement, based on (estimated) patient seroconversion dates from serotesting at regular intervals.
- Quantification of RNA was done using a Reverse Transcriptase-Polymerase Chain Reaction assay, with a lower detection limit of less than 1000 copies/mL. Both serum and plasma measurements were included, as these differ only by a relatively small amount compared to variations over time and between individuals [18], [19].
- For prognostic studies, median follow-up was more than 1 year.
- Study subjects were not treated with triple-therapy ART or any antiretroviral combination containing protease inhibitors. Studies that included patients receiving zidovudine monotherapy or two nucleoside analogues (for subgroups of participants, during part of follow-up) were included, because these regimens do not significantly affect RNA or CD4 levels over several years [5], [8], which was the typical length of follow-up in prognostic studies analyzed – and two studies reported no influence of non-triple ART use on RNA and CD4 prognostic value [20], [21].

### Within-population variability in RNA and CD4

For within-population variability in RNA and CD4 levels, eligible studies had to report at least two statistics of: median, mean, IQR or other centiles, minimum, maximum and standard deviation. We standardized variability statistics across all studies as medians with IQRs, deriving missing medians and IQRs by applying normal distributions on transformations of the reported alternative statistics.

Appropriate transformation functions that normalized RNA and CD4 distributions were identified by analysis of raw data from a cross-sectional survey of HIV-infected South-African men [22]. A Box-Cox transformation [23], that minimizes the skewness of the frequency distribution, was found to produce adequate normal distributions:
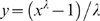
where x is the untransformed value of RNA viral load (copies/mL) or CD4 (cells/µL) and *y* is the corresponding transformed value. Estimated values of *λ* that fitted the South African data were 0.1606 for RNA and 0.5420 for CD4. The same power law and values of *λ* were then used to derive RNA and CD4 medians and IQRs from studies not reporting these statistics, by fitting cumulative distribution functions [24].

### Relative prognostic risks associated with RNA and CD4

The analysis of relative risks associated with high RNA and low CD4 focused on the two outcome measures AIDS and death. Standard definitions of AIDS (group C of the Centers for Disease Control and Prevention 1993 [25] or 1986/7 [26], or European 1993 [27] classification) were accepted. Where available, we included the reported univariate relative risks per 100 cells/µL lower baseline CD4, and per 10-fold (1 log10/mL) higher baseline RNA. Univariate risks were felt to best reflect the value of simple, individual predictor variables as used in clinical practice (where e.g. CD4 thresholds are constant irrespective of patients' RNA, age and sex). For studies reporting only multivariate risks (adjusted for covariates), however, those measures were included.

To capture the maximum possible information from relevant studies despite limitations in data and variability in risk measures reported (Table 1), we proceeded as follows:

- Studies which reported relative risks only for quartiles of RNA or CD4 were included, assuming that each successive quartile approximated a 10-fold higher RNA or a 100 cells/µL lower CD4 – as was the case in one study that reported on both risk measure units [21].
- Where progression risk was reported as ‘non-significant’ without specification of the exact value, this was entered as 1.0.
- From studies that reported relative risks for RNA or CD4 ‘baseline’ measurements taken at two or more different timepoints relative to seroconversion [4], [28], [29], all estimates were included, to capture all information on the development over time in the prognostic marker.
- From studies that reported risks over two or more different durations of follow-up (from a fixed timepoint of baseline RNA or CD4 measurement) [30], [31], we included the estimate for the shortest follow-up duration, as short-term prognostic indications are likely to be most relevant in patient management.
- For studies of seroprevalent cohorts for whom no (estimated) median time interval between seroconversion and (baseline) RNA or CD4 measurement was reported, this timepoint was imputed based on the reported baseline CD4 level and the relationship between the measurement timepoint and baseline CD4 in studies that reported both.

**Table 1:** Studies of relative risks of AIDS and/or death associated with higher RNA viral load or lower CD4 cell count, in populations of HIV-1 infected adults.

Setting (cohort)	Population	N	Baseline year£		Follow-up+	Annual pro-gression to:		ARV mono-/bi-therapy		RNA prognostic risk*:			CD4 prognostic risk*:		
				CD4		AIDS	Death	Baseline	% of follow-up	Unit RNA increase	AIDS	Death	Unit CD4 decrease	AIDS	Death
NY City, USA[21]	F general population	1769	4.4££	n.a.	1.5		9.5%	64% of participants	13%	10-fold		2.2 (adjusted)♣	Quartile		3.1♣ #
Washington DC & NY City, USA [19]	MSM	111	1.5££	n.a.	5.0	12%	12%	No	23%&	3-fold	1.9	1.6			
Zimbabwe [67]	General population	196	4.4££	330	3.6		8.0%	No		0.1 log10		1.2	100 cells/µL		1.7
Gambia [90]	Pregnant F	101	2	n.a.	6.9		4.6%	No		10-fold		1.8 (adjusted)			
Mombasa, Kenya [73]	F sex workers	218	1.2	498	4.6		4.0%	No		10-fold		2.2	100 cells/µL		1.4
Italy [75]	IDU, MSM and heterosexual SC	86	0.7	619	4.8	4.8%		No	35%	10-fold	1.8		100 cells/µL	1.0	
USA (MACS) [7]	MSM	218	0.25	741	4.0	17%			32%&	Quartile	1.6#				
			0.75	599	4.0	16%				Quartile	1.7#				
			1.25	565	4.0	17%				Quartile	1.9#				
			1.75	503	4.0	17%		No		Quartile	2.0#				
USA (MACS) [20]	AIDS-free MSM	1416	2.1££	500	6.7	9.2%	7.9%	No	32%& ♦	Quartile	2.0#	2.1#	Quartile	1.5#	
Denmark [31]	M SC	93	1.2	480	6.0	5.7%		3%	28%&	Quartile	2.1#		Quartile	2.8#	
France (SEROCO) [28]	SC MSM, heterosexual M+F and IDU	271	0.9	534	7.0	4.4%	3.3%	No	20%&	10-fold	3.0	2.9	100 cells/µL	1.3	1.3
		112	0.3	594	6.3	4.3%	2.3%				2.1	2.6		1.1	1.0
NY city, USA [30]	AIDS-free MSM	150	1.0££	625	2.0	29%		No		Quartile	2.5#		Quartile	1.9#	
Abidjan, Cote d'Ivoire [91]	SC blood donors	104	0.8	527	2.0	1.9%	1.0%	No		10-fold	3.2			1.0$	
Baltimore, USA [84]	AIDS-free IDU	522	2.0££ +	510	6.4	4.4%	3.6%	6% of participants	25%&	3-fold	1.3	1.4	100 cells/µL	1.2	1.1
Amsterdam, Netherlands [4]	MSM	119	0.5	689	5.6	7.9%		No	27%&	Quartile	1.0$				
		117	1	641	5.3	8.3%				Quartile	1.9#				
		105	3.5	375	4.1	11%				Quartile	2.7#				
		117	1	641	5.3	8.3%		No	27%&				n.a.	1.0$	
		105	2	509	4.8	9.1%							Tertile	2.1#	
		105	5	327	3.3	13.3%							Tertile	4.5#	
		105	6	348	2.7	16%							Tertile	3.2#	
Switzerland [85]	IDU, MSM, heterosex-uals and others	394	6.1££	330	2.4	15%	18%	No	28%&	Quartile	1.9#	2.5#	Quartile	2.0#	3.0#
Denmark & USA [92]	MSM	201	1.0££	n.a.	5.0	11%		3%	28%&				100 cells/µl	1.6	
UK (CHIC) [93]	All risk groups, 72% MSM, 20% hetero-sexuals, 16% F	11469	1.63££	536	1.7		0.24%	No					100 cells/µl		1.4

Median durations of follow-up across studies and datapoints were 4.8 years for AIDS depending on RNA; 6.3 years for death depending on RNA; 4.9 years for AIDS depending on CD4, and 4.6 years for death depending on CD4.

ARV = antiretroviral; MSM = men having sex with men; IDU = injection drug users; F = women; M = men; RR = relative risk; SC = seroconverter or seroconverting.

* Unadjusted, unless indicated.

# For studies reporting relative risks per quartile or tertile, the value stated is the average relative risk over the quartiles (Q2/Q1, Q3/Q2, Q4/Q3) or tertiles (T2/T1, T3/T2).

$ 3 studies that reported a relative risk as ‘not significant’ without specifying the risk value were entered at a value of 1.0. Omission of these 3 datapoints did not significantly alter the qualitative or quantitative results or conclusions (not shown).

£ HIV infection/disease stage at baseline RNA or CD4 measurement, as the median year after SC in the study population.

££ Baseline year not reported, but imputed from baseline CD4 level by extrapolation of the linear relationship between baseline CD4 and baseline year from the 14 studies that reported both (Pearson's R^2^ = 0.72, p<0.001).

+ Median duration of patient follow-up, in years.

& For studies reporting only the proportion of participants who received any antiretroviral mono- or bi-therapy during follow-up but not the proportion of follow-up duration affected by therapy, we estimated the latter as half the proportion of participants who received any therapy.

♣ RRs similar when including only women who had not received ART, by censoring follow-up at ART initiation, or by including ART use as time-varying covariate.

♦ Baseline RNA levels similar in subgroups that did and did not receive ART during f-up; RNA predicted time to AIDS and death independent of subsequent ART.

The dependence of risks on timepoint of RNA/CD4 measurement, duration of follow-up, average rate of progression to AIDS or death during follow-up, median baseline CD4 in the study population, geographical region and use versus non-use of antiretroviral mono- or bi-therapy at baseline and during follow-up was assessed in linear regression. All studies were weighted equally, because heterogeneity in risks due to factors such as study design and follow-up, setting, clinical spectrum of patients at baseline and median progression rates was much greater than sampling errors. Factors significant at p<0.10 in univariate analysis were considered for multivariate models.

### Components of within-population variability

As a complementary indication of the population-level prognostic values of RNA and CD4 measurement, we quantified the extent to which within-population variation in each measure reflects within-individual variation, due to laboratory measurement error and/or short-term physiological fluctuation – which confounds prognostic value – versus variation among individuals – which determines and constitutes clinical prognostic value.

Relevant studies reporting components of within-individual variability for both RNA and CD4 from the same set of patients were identified by searching PubMed. Their results were analyzed in the light of total within-population variations aggregated from the larger set of studies reporting the latter.

## Results

### Within-population variability in RNA and CD4

**Figure 1:**
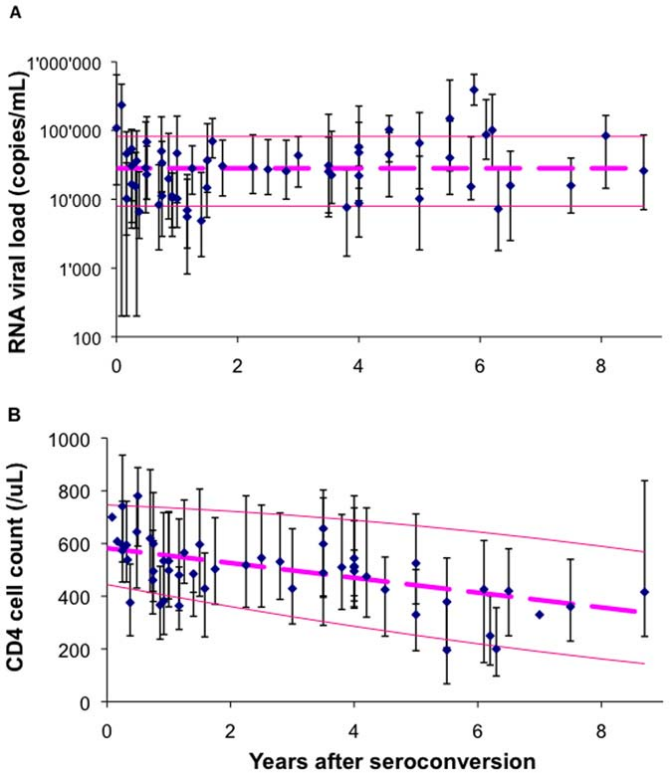
(A) RNA viral load; (B) CD4 cell counts, over the course of untreated HIV-1 infection in adults. Each blue dot represents one datapoint of a median RNA or CD4, with the horizontal error bar indicating the corresponding interquartile range in the study population. Bold pink lines are medians across all population medians, for which thin pink lines indicate the corresponding interquartile range. Data sources: (a) [6]–[8], [11], [28], [29], [31], [66]–[85]; (b) [7], [8], [11], [28], [29], [31], [66]–[71], [73]–[88]. The (linear) trends over time since seroconversion in population median RNA and CD4 should not be interpreted as a proxy of trends in RNA and CD4 in individual patients. Since population medians are conditional on patients being alive, in individual patients RNA will instead tend to increase over time, and CD4 will tend to decrease stronger than apparent from Figure 1b.

RNA medians with IQRs at a specified (estimated) time after seroconversion were available from 57 measurements (Figure 1a). Across these data, population medians varied widely, between 5,000 and 390,000 copies/mL. The IQR averaged around 74,000 copies/mL, or 2.5 times the population median. For the overall median across studies of 28,000 copies/mL the corresponding IQR would be ≈8,000–82,000. In linear regression, the IQR did not change significantly with the timepoint after seroconversion (p = 0.37), but increased with increasing population median RNA (p<0.001). When expressed as a proportion of population median value, the IQR did not vary with time (p = 0.64) or with median RNA value (p = 0.052).

For CD4, across 51 datapoints, population medians decreased over time after seroconversion, from around 600 cells/µL in the first year to 360 cells/µL for populations sampled at around 8 years after seroconversion (Figure 1b; p for trend<0.001). Corresponding IQRs averaged 330 cells/µL, or 0.65-fold the population median. For the overall median of 500 cells/µL the corresponding IQR would be 360–700 cells/µL. In linear regression, the IQR did not vary significantly with either the population-specific median CD4 or the timepoint after seroconversion (p = 0.11 and 0.095, respectively). When expressed as a proportion of population median, however, the IQR increased with time after seroconversion (p<0.001; Figure 1b).

### Relative prognostic risks per unit RNA or CD4 difference

**Figure 2:**
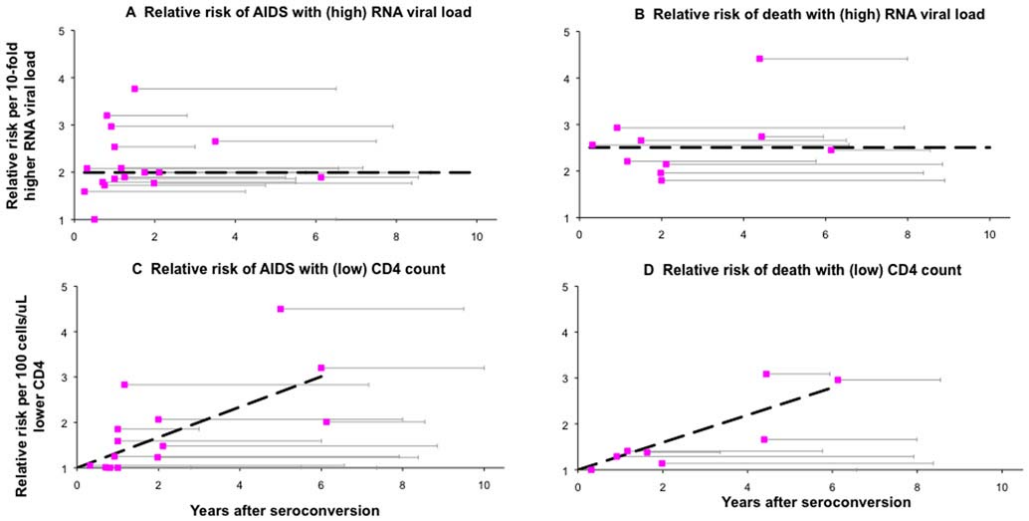
Relative risks of clinical HIV progression per unit difference in RNA or CD4. A. Risk of AIDS per 10-fold (1 log10/mL) higher RNA; B. Risk of death per 10-fold (1 log10/mL) higher RNA; C. Risk of AIDS per 100 cells/µL lower CD4; D. Risk of death per 100 cells/µL lower CD4. Each symbol represents the estimate from 1 study, of a population of HIV-1 infected adults (see Table 1 for details of studies). Risks are displayed as a function of the median time since HIV seroconversion that RNA or CD4 was first measured. Horizontal error bars indicate the median duration of follow-up over which RR was evaluated. Dashed lines in (a) and (b) indicate pooled median RRs across studies, which did not vary with time since seroconversion. Dashed lines in (c) and (d) indicate linear trends of increasing RR with stage that CD4 was measured (from a defined value of 1.0 at seroconversion; c: Pearson's R^2^ = 0.74; p<0.0001; d: Pearson's R^2^ = 0.89; p<0.0001). Median follow-up across studies and datapoints were (a) 4.8 years; (b) 6.3 years; (c) 4.9 years, (d) 4.6 years. If instead of univariate relative risks, multivariate relative risks were preferentially included from studies that reported both, results did essentially not change (a: 6 studies reporting both RRs, with median ratio of multivariate-to-univariate RR 0.82; b: 6 studies reporting both, median ratio of multivariate-to-univariate RR 0.84; c: 4 studies reporting both, median ratio of multivariate-to-univariate RR 0.94; d: 5 studies reporting both, median ratio of multivariate-to-univariate RR 0.91).

Mean relative risks per 10-fold higher RNA were 2.0 (95% confidence interval (CI): 1.8–2.5) for AIDS (12 studies, 17 datapoints) and 2.5 (2.1–3.0) for death (9 studies, 10 datapoints; Table 1). These prognostic risks did not vary over time after seroconversion (Figure 2a and 1b), or with duration of follow-up, geographical region, baseline CD4, use of antiretroviral mono-/bi-therapy, or average clinical progression rates (see Text S2).

In contrast, relative risks per 100 cells/µL lower CD4 increased with time after seroconversion, for both AIDS (10 studies, 14 datapoints) and death (7 studies, 8 datapoints; Table 1) (Figures 2c and 1d): from 1.0 at seroconversion to an estimated 3.0 (95% CI 2.6–3.4) by 6 years for AIDS (p<0.0001 for trend), and to 2.8 (1.9–3.7) for death (p<0.0001). In multivariate regression, this pattern was not modified by duration of follow-up, geographical region, baseline CD4, use of antiretroviral mono-/bi-therapy or average progression rates (see Text S2).

### Prognostic power at population level

**Figure 3:**
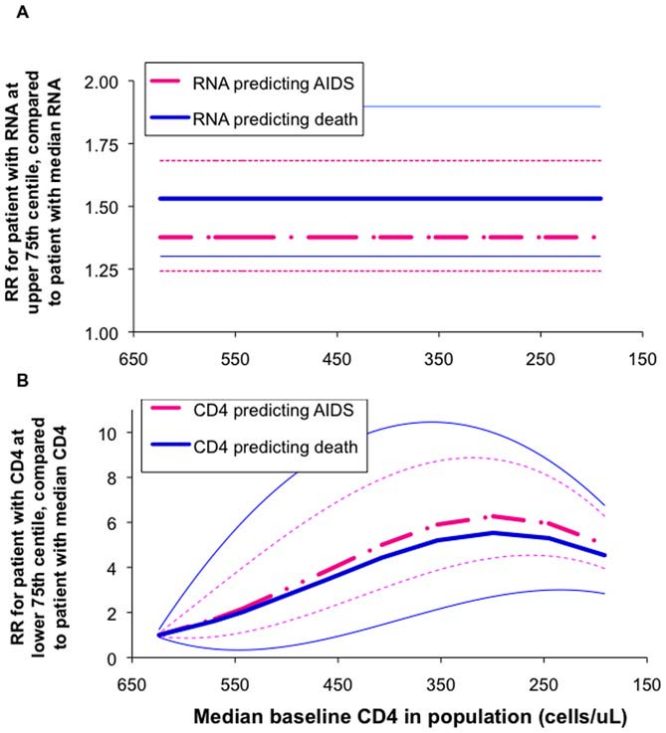
Population-level prognostic power of RNA and CD4 in untreated HIV-1 infection. A. Relative prognostic risk (RR) for a typical patient at 75^th^ centile highest RNA, compared compared to the average patient with exactly the population-median RNA value; B. Relative prognostic risk for a typical patient at 75^th^ centile lowest CD4, compared compared to the average patient with exactly the population-median CD4 value. Results are expressed as a function of median CD4 in the population, for the range of median CD4 levels found in studies analyzed in Table 1 and Figure 1. CD4 population medians were calculated as a linear function of the median year after seroconversion, based on the studies presented in Table 1. Bold lines indicate best estimates; thin lines 95% confidence intervals.

When combining the results from Figures 1 and 2, population-level prognostic powers can be approximated as the relative risk for a hypothetical patient with RNA at the upper 75^th^ centile or CD4 at the lower 75^th^ centile, compared to a patient with exactly the median RNA or CD4 level in a given population (Figure 3).

Whereas relative risks per 10-fold RNA (multiplicative) increase were similar to relative risks per 100 cells/µL (linear) CD4 decline (Figure 2), the population-level prognostic value was then considerably higher for CD4 than for RNA, except for populations limited to very recently infected people. For RNA, relative prognostic risks for a patient with viral load at upper 75^th^ centile were a modest 1.4 (95% CI 1.2–1.7) for AIDS and 1.5 (95% CI 1.3–1.9) for death, compared to a patient with median RNA. These values were constant across the range of population baseline CD4 medians (Figure 3a).

For CD4, in contrast, the prognostic risk at the lower 75^th^ centile compared to the ‘median’ patient, rose markedly with decreasing median CD4: from 1.0 (i.e., no prognostic information) at CD4s above 625/µL – which, in our dataset, corresponded to the time of seroconversion – to maxima of 6.3 (95% CI 4.4–8.9) for AIDS and 5.5 (95% CI 2.7–10.1) for death in populations with median 300 CD4/µL – which, in the dataset of prognostic risks, corresponded to around 6 years after seroconversion (Figure 3b).

The time pattern of increasing CD4 prognostic risks was stronger at a population level (Figure 3b) than at individual-level (Figure 2c&d), because the (proportional) population-level variability in CD4 increases over time after infection (Figure 1b). Below a population median of 300 cells/µL (around year 6 after seroconversion), the absolute CD4 range within populations started to decrease (despite continued increase in proportional variability). As a result, population-level prognostic value tended to fall again, although this decline did not reach statistical significance within the CD4 range evaluated (down to 190 cells/µL; Figure 3b). In intuitive terms: by the time that most or all patients have progressed to low CD4, the prognostic value of CD4 will start to decrease.

### Components of within-population variability

One study reported components of within-population variability for plasma RNA and CD4 simultaneously [32], permitting a comparison of variability components between the indicators. In this study, 30 clinically stable seropositive men in the UK receiving antiretroviral mono-therapy had blood samples taken for RNA and CD4 measurement at 6 occasions spread over 6 months. Each sample was analyzed twice. Differences between duplicate assays were considered to reflect laboratory/measurement error, while variation between visits within a patient was attributed to physiological fluctuation.

For RNA, among patients with enrollment levels of >500/mL, the median within-patient range of values across 6 measurements was ≈6,500 copies/mL (0.76 log10), while the median difference between duplicate assays was ≈1,500/mL (0.2 log10). For CD4, the median within-patient range over 6 months was 119 cells/µL, and the median difference between duplicate assays 16 cells/µL. Using components of variance analysis, the authors estimated that for RNA, physiological and measurement factors accounted for 55% and 45% of variation within individuals, while corresponding contributions were 92% and 8% for CD4 [32].

**Figure 4:**
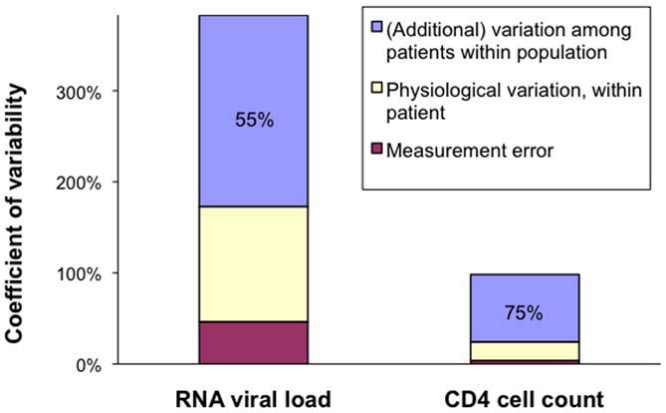
Within-population variability in RNA and CD4 during untreated adult HIV-1 infection, by component of variation. ‘Coefficients of variability’ were defined and calculated as described in Table 2. The percentages written in the blue bars indicate the proportion of overall variability that is *not* attributable to within-patient factors.

Figure 4 shows how this within-individual variability contributes and compares to overall within-population variability. Outcomes for all three variability components (among individuals in a population, within-individual physiological and within-individual laboratory measurement error) were standardized as ‘coefficients of variability’, relative to the corresponding population median values (Table 2). For RNA, total within-individual variation was thus estimated to make up about 45% of within-population variation. For CD4, within-individual variation would account for around 25% of within-population variation. The corresponding estimated proportion of variation *among* individuals is therefore smaller for RNA than for CD4 (55% and 75%, respectively: blue areas in Figure 4), reinforcing the conclusion that, among patients in the chronic stage, CD4 has better prognostic power than RNA.

**Table 2:** Definition and calculation of components of within population-variability in RNA viral load and CD4 cell count during untreated HIV-1 infection in adults.

Component of variation		Definition of ‘Coefficient of Variability’	Data sources	Coefficient of variability (calculation)	
				RNA (copies/mL)	CD4 (cells/µL)
Measurement error		Median difference between duplicate assays within a patient visit, as proportion of population median value	[32]	46% ( = 1,538/3,311*)	4% ( = 16/400)
Physiological variation		Within-individual variation minus measurement error, as proportion of population median value	[32]	126% ( = 173%–46%)	20% ( = 24%–4%)
	} (total) Within-INDIVIDUAL variation	Median 95% range of values within a patient obtained over 6 visits, as proportion of population median value	[32]	173% ( = 5,717/3,311*)	24% ( = 98$/400)
Additional variation among individuals		Within-population variation minus within-individual variation, as proportion of population median value	Studies of Figure 1 +; [32]	210% ( = 383%–173%)	74% ( = 98%–24%)
	} (total) Within-POPULATION variation	Median within-population IQR divided by 0.675#, as proportion of population median value	Studies of Figure 1 +	383% ( = (73,600/0.675#)/28,200)	98% ( = (330/0.675#)/500)

* Samples with RNA>500 copies/mL only. The 95% confidence interval was calculated based on the reported standard deviation among 6 visits, of 0.264 log10 copies/mL, applying a Student T-statistic for 5 degrees of freedom.

$ Lacking a reported standard deviation, the 95% confidence interval was estimated by multiplying the reported full ( = 119 cells/µL) CD4 range among 6 visits with the ratio of 95% range to full range ( = 5,392/6,563 copies/mL) in RNA.

# For a normal distribution, the 95% confidence interval is equal to the interquartile range divided by 0.675. As population median value, we here take the median across all studies shown in Figure 2.

+ Average within-population variability was taken from the pool of eligible studies depicted in Figure 1, as the sample in [32] was too small to provide a representative estimate of typical within-population variability.

## Discussion

This quantitative review confirmed that RNA and CD4 have very different time patterns of clinical prognostic value during untreated HIV-1 infection [4].

Within the first 2 years of infection, RNA immediately gives some indication of long-term prognosis. Due to constant relative risks (Figure 2a&b) and constant within-population variability (Figure 1a), RNA remains similarly informative when measured during later years (Figure 3a). CD4, in contrast, carries little prognostic value over early years. Its within-population variability then instead largely relates to pre-infection CD4 levels, which vary by up to a factor ten among uninfected adults without influencing prognosis after infection [10]. As infection progresses and worsening immune deficiency allows opportunistic infections (OI) and AIDS-defining illnesses to occur, the prognostic value of CD4 increases (Figure 3b), due to strong increases in relative prognostic risks per unit CD4 decrease (Figure 2c&d) and increasing proportional within-population variability in CD4 levels (Figure 1b). Part of the prognostic value may reflect that OI themselves temporarily reduce CD4 [33] and increase RNA [34], [35] − effects that may be common in clinic populations where the occurrence of OIs is often the reason for diagnosis, especially in Africa. The pattern of diminishing CD4-related progression risks at higher baseline CD4 (Figure 2c+d) was also observed in a recent multi-center analysis of patients starting immediate versus deferred ART [36]: Here, the prognostic value of CD4 − as measured by AIDS/death risks associated with deferring ART − was high at low CD4 thresholds, but marginal at thresholds in the range of 500 cells/µL.

**Figure 5:**
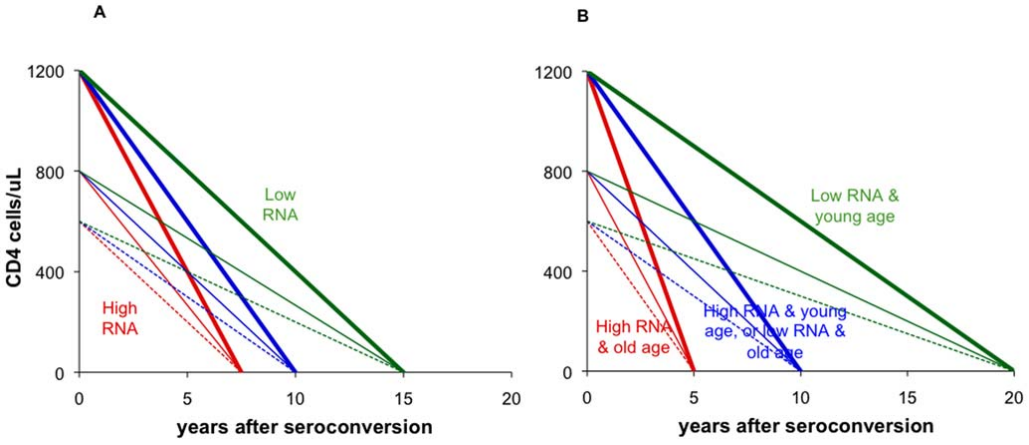
Schematic natural history model of HIV-1 replication driving rates of CD4 decline and clinical progression. A. Survival varies among individuals according to the level of viral replication – which is indicated by RNA after reach of the setpoint in the first months after seroconversion: patients with highest RNA (red lines) have shortest survival; patients with lowest RNA (green lines) have longest survival. Rates of CD4 decline varies according to (1) RNA setpoint; and (2) pre-infection CD4, which varies independently without influencing prognosis upon infection. Individuals with high CD4 before infection have faster subsequent CD4 decline (bold lines) than individuals with low pre-infection CD4 (dashed lines) – for a given RNA and duration of survival. This natural history model has earlier been proposed based on data of cohorts of homosexual men in New York city and Washington DC [89]. B. Refinement of natural history model to incorporate prognostic determinants not (or not entirely) operating through RNA and CD4; these increase the within-population variability in survival (x-axis range). ‘Age’ here could symbolically be taken to also stand for other factors independently associated with prognosis: e.g. good immune constitution at baseline or low exposure to pathogens causing opportunistic infections, instead of young age.

Within 2 years after seroconversion, CD4 surpasses RNA as a prognostic marker, and in later years its prognostic value far exceeds that of RNA (Figure 3). This estimation supports the natural history model whereby viral replication drives CD4 decline and clinical progression (Figure 5a). The marked superiority of CD4 as prognostic marker during untreated infection is furthermore supported by its estimated near 2-fold smaller proportional laboratory measurement error and physiological fluctuation within patients, compared to RNA (Figure 4).

### Implications for ART initiation and allocation

As of 2008, the common protocol in USA and Western Europe is to initiate treatment when CD4 falls below 350/µL or in case of a history of an AIDS-defining illness [1]. For low-income countries, as a ‘public health approach’ the WHO recommends ART for people in WHO clinical stage IV regardless of CD4, consideration of treatment in stage III with CD4 below 350/µL, and treatment for those with CD4 below 200/µL regardless of clinical stage [3]; determining viral load is unnecessary. Our results support the WHO recommendation to start antiretroviral treatment in low-income settings based on clinical criteria supplemented with CD4, without pre-treatment RNA monitoring − if the purpose of ART is to maximize clinical benefit among patients for given treatment and diagnostic inputs. By revealing that prognostic power is highest in populations with median CD4 around 300/µL (Figure 3b), findings furthermore assist in interpreting recommendations for how to manage treatment-naïve individuals with CD4 between 200–350/µL − an area of therapeutic uncertainty.

Simulation studies that combined assumed CD4 and RNA prognostic risks and within-population variabilities with observed clinical responses to ART in different CD4 and RNA strata also found that application of CD4 thresholds to supplement clinical criteria improves the effectiveness and cost-effectiveness of ART [37]–[39]. An observational study of South African patients added the *proviso* that clinical criteria alone are a better predictor of disease progression and ART benefit than CD4 alone [40]. Studies evaluating different CD4 thresholds predicted that thresholds above 350 cells/µL would save more lives and life-years, but at lesser cost-effectiveness [38], [41] − with routine start at CD4<350/µL being cost-effective by international economic standards [42].

Regarding the utility of RNA measurement, two models comparing CD4-based and RNA-based treatment initiation concluded that, to scale-up ART beyond symptomatic patients presenting in clinics, combined RNA/CD4 algorithms would be most efficient [43], [44]. In contrast, a comparison of clinical, CD4- and RNA-based screening algorithms for a population in Côte d'Ivoire found RNA testing to be of high cost and dubious additional benefit [45], and another simulation predicted low cost-effectiveness for adding RNA measurement to clinical criteria and CD4 testing [39].

Most simulation models assumed a fixed increase in risk of clinical progression per unit CD4 decline during untreated infection, throughout the spectrum of CD4 levels and infection stages. This simplistic assumption is at odds with the progressive increase in relative hazard with decreasing CD4 stratum (i.e. increasing duration of infection) that emerged from our analysis (Figure 2c&d). Furthermore, only one model [38] incorporated random fluctuation in CD4 (or RNA) measurement in simulations. If accounting for both the time-changing CD4-related risk and dilution of prognostic value due to within-individual fluctuations that our analyses quantified, these models would probably predict an even lower cost-effectiveness for applying CD4 thresholds above 350 cells/µL and for RNA pre-treatment monitoring.

### Implications for screening strategies for ART-naïve people

The substantial within-patient fluctuation for both CD4 and RNA implies that prognostic value can be improved by repeating blood sampling and measurements within patients, and evaluating their average levels over time. Pooling repeat values would probably increase prognostic risks relative to those shown in Figures 2&3, which mostly derived from single RNA or CD4 measurements.

Modelling studies have calculated, as the most efficient CD4 testing algorithm for ART-naïve people, a default measurement frequency of not more than once yearly [44], with repeat testing for patients whose initial value is close above the treatment threshold (e.g. between 350–400 cells/µL) [38], [41]. Stratification by age, with more frequent tests for older people in whom CD4 declines faster, would further increase efficiency [38], [41].

### Limitations

The analyses have several limitations. First, the majority (∼75%) of eligible studies were from high-income, Western settings – leaving the applicability of findings uncertain especially in Africa.

For relative risk studies, data analyzed were limited to untreated cohorts and did not address toxicity, viral resistance and cost associated with (early versus late) ART. We nevertheless believe that the findings are important for clinical decision making, because the prior question that physicians face is their patients' prognosis if treatment is *not* initiated. Furthermore, despite our attempts to include only high-quality studies and to focus on standardized outcomes with known covariates, our pooled analyses are not meta-analyses in the strict sense. Notably, included studies varied in rates of loss to follow-up, extent of exposure to antiretroviral mono- or bi-therapy, OI prophylaxis and treatment, and patient inclusion criteria and age ranges; however, available (cohort-aggregate, rather than individual-patient) data and statistical power precluded optimal assessment of these possible determinants (see Text S2).

Although one would ideally like to know *absolute* risks of AIDS and death, these are both highly variable between patients and uncertain. Therefore, treatment guidelines must be based on population estimates of *relative* risks for different categories of patients, such as we provide.

Analyses focused on RNA and CD4 as independent individual markers, ignoring non-laboratory-based markers that are typically more accessible, at lower cost, for larger groups of patients. Notably, age at initial infection is an important determinant of survival [46]–[48], which in part underlies the effect of RNA and CD4: older patients have higher viral setpoints [49] and hence faster CD4 decline and disease progression (Figure 5b). Since age at initial infection or its approximation, ‘age at first clinical presentation’, will be available for all patients even in the most peripheral facilities, the most relevant question to answer would be the prognostic value of RNA and CD4 *additional to* age (and clinical and other socio-demographic factors) [47]. Published reports did not allow such analysis, but this will be eagerly awaited from multicentre patient cohorts such as CASCADE, ART-LINC and ICONA [47], [50], [51].

When considering expansion of CD4-guided treatment initiation, thresholds may furthermore need to be differentiated between populations, to account for geographical and age/sex differences in pre-infection distributions [10]. For example, in populations with low CD4 levels in both HIV-infected and HIV-uninfected adults, such as Ethiopians [52] and Chinese [53], thresholds above 350 cells/µL would probably be (even) less efficient than in other populations.

In the analysis of within-population variabilities, we did not attempt to explain variation among studies in *median* marker levels beyond that explained by stage of infection (Figure 1). The wide variation in population medians for both RNA and CD4 is itself a significant observation, which may justify further investigations into geographical and socio-demographic patterns – as well as reflection on the validity of universal, international reference values and treatment thresholds [10].

The analysis of components of within-population variability in CD4 and RNA (Table 2 and Figure 4) was based on the findings of just one 30-patient dataset [32]. No other studies were identified that estimated the contributions of measurement error and physiological fluctuation for both RNA and CD4 in parallel. However, several studies that measured a subset of these outcomes supported the findings of study [32], namely:

- RNA measurements fluctuate within patients over short periods of time by 0.2–0.5 log10 copies/mL [54]–[58], with measurement error and physiological fluctuation (without a diurnal pattern) each contributing about half to this variation [54], [55], [58]–[60];
- CD4 measures vary within patients by 60–130 cells/µL within weeks, with a larger contribution of physiological (diurnal) fluctuation than of measurement error [56], [61], [62];
- Proportionally, measurement error and overall within-individual variation are larger at lower median CD4 [56] and RNA [32], [63];
- During stable infection, overall within-subject variation is proportionally larger for RNA than for CD4 [57].

While these analyses help to better understand the respective value of RNA and CD4 measurements in decision making for treatment-naïve patients, biomarker testing and treatment allocation policies should ultimately reflect not just knowledge of HIV natural history and clinical prognosis, but a broader societal debate about the individual-level and public health objectives of ART [16]. By end-2007, in low- and middle-income countries an estimated 31% (2.9 out of 9.7 million) of people in need were getting ART [14]. As long as resources remain insufficient to treat all people in need, prioritization among patients must inevitably consider not only the individual patient's health gain, but also the broader public health objectives: Is treatment meant to maximize patients' survival (for given treatment and diagnostic inputs)? To restore workforce capacity, and/or to prevent orphaning and reduce socio-demographic impact of HIV/AIDS? To contribute to HIV prevention, by lowering viral load and reducing transmission to sexual partners of patients? Different objectives would guide treatment priorities in different directions: for example to the sickest and oldest, to employees in critical economic sectors and young parents [64], or to the most sexually active (risk) groups and/or patients with highest RNA [65].

### Conclusions

Although high RNA is the earliest predictor of clinical progression after HIV seroconversion, for populations of treatment-naïve patients during stable infection or with unknown date of seroconversion, CD4 surpasses RNA as a prognostic indicator and screening tool to select patients in need of ART. The prognostic value of CD4 in treatment-naïve patients was shown to be greatest in populations with median ≈300 cells/µL. Findings support the WHO recommendation to start antiretroviral treatment in low-income settings based on clinical criteria supplemented with CD4, without pre-treatment RNA monitoring. Whereas expanding CD4 treatment initiation thresholds to above around 350 cells/µL would increase the life-years gained with ART, the added value of CD4 measurement then diminishes. A public health approach to expanding ART allocation in low-income countries may benefit from differentiating or supplementing CD4 thresholds by independent socio-demographic or public health prognostic indicators, such as age.

## Supporting Information

Text S1 MOOSE Guidelines for meta-analysis and systematic reviews of observational studies – with specification of adherence of literature searches, study selection and analyses conducted.
(0.14 MB DOC)

Text S2 Statistical Appendix. Univariate and multivariate regressions of relative risks of clinical HIV progression associated with higher RNA viral load and lower CD4 cell count.
(0.08 MB DOC)
