# Supplementary material for: Clinical Prognostic Value of RNA Viral Load and CD4 Cell Counts during Untreated HIV-1 Infection—A Quantitative Review
Source: PLoS One. 2009 Jun 17;4(6):e5950. doi: 10.1371/journal.pone.0005950 (PMC2694276; doi:10.1371/journal.pone.0005950)
Supplement: Text S1 — MOOSE Guidelines for meta-analysis and systematic reviews of observational studies – with specification of adherence of literature searches, study selection and analyses conducted. (0.14 MB DOC) [file pone.0005950.s001.doc]

**Text S1**

Supporting Information to article ‘*Clinical prognostic value of RNA viral load and CD4 cell counts during untreated HIV-1 infection’*, by Korenromp-EL *et al.*

**MOOSE Guidelines for Meta-Analyses and Systematic Reviews of Observational Studies***-- with specification of adherence of literature searches, study selection and analyses conducted.

***Title*** Identify the study as a meta-analysis (or systematic review) √

***Abstract*** Use the journal’s structured format √

***Introduction***

**Present**:

• The clinical problem √

• The hypothesis √

• A statement of objectives that includes the study population, the condition of interest, the exposure or intervention, and the outcome(s) considered √

***Sources***

**Describe**:

• Qualifications of searchers (eg, librarians and investigators) √ (Literature searches were conducted by principal investigator EK)

• Search strategy, including time period included in the synthesis and keywords √

• Effort to include all available studies, including contact with authors √

• Databases and registries searched n.a.

• Search software used, name and version, including special features used (eg, explosion) √

• Use of hand searching (eg, reference lists of obtained articles) √

• List of citations located and those excluded, including justification We did not keep track of the (hundreds of) abstracts and articles screened and excluded.

• Method of addressing articles published in languages other than English √

• Method of handling abstracts and unpublished studies √

• Description of any contact with authors √

***Study Selection***

**Describe** **:**

• Types of study designs considered √

• Relevance or appropriateness of studies gathered for assessing the hypothesis to be tested: √

(Implied in selection of eligible studies)

• Rationale for the selection and coding of data (eg, sound clinical principles or convenience): √

(Implied in eligibility criteria)

• Documentation of how data were classified and coded (eg, multiple raters, blinding, and interrater reliability) √

• Assessment of confounding (eg, comparability of cases and controls in studies where appropriate): n.a. (Absence of confounding on relevant outcome measures implied in eligibility criteria)

• Assessment of study quality, including blinding of quality assessors; stratification or regression on possible predictors of study results √

• Assessment of heterogeneity √

• Statistical methods (eg, complete description of fixed or random effects models, justification of whether the chosen models account for predictors of study results, dose-response models, or cumulative meta-analysis) in sufficient detail to be replicated √

***Results***

**Present**:

• A graph summarizing individual study estimates and the overall estimate √

• A table giving descriptive information for each included study √

• Results of sensitivity testing (eg, subgroup analysis) √ (Included as regression analyses, in as Supplementary material #2: ‘Statistical Annex’)

• Indication of statistical uncertainty of findings √

***Discussion***

**Discuss**:

• Strengths and weaknesses √

• Potential biases in the review process (eg, publication bias) √

• Justification for exclusion (eg, exclusion of non–English-language citations) Implied in eligibility criteria

• Assessment of quality of included studies: √ (No explicit assessment, but minimum quality implied in eligibility criteria)

• Consideration of alternative explanations for observed results √

• Generalization of the conclusions (ie, appropriate for the data presented and within the domain of the literature review) √

• Guidelines for future research √

• Disclosure of funding source √

*Modified from Stroup DF, Berlin JA, Morton SC, Olkin I, Williamson GD, Rennie D, et al. *Meta-analysis of observational studies in epidemiology: a proposal for reporting. Meta-analysis Of Observational Studies in Epidemiology (MOOSE) group.* *JAMA* 2000;**283**:2008–12. Copyrighted © 2000, American Medical Association. All rights reserved.
