# Supplementary material for: Clinical Prognostic Value of RNA Viral Load and CD4 Cell Counts during Untreated HIV-1 Infection—A Quantitative Review
Source: PLoS One. 2009 Jun 17;4(6):e5950. doi: 10.1371/journal.pone.0005950 (PMC2694276; doi:10.1371/journal.pone.0005950)
Supplement: Text S2 — Statistical Appendix. Univariate and multivariate regressions of relative risks of clinical HIV progression associated with higher RNA viral load and lower CD4 cell count. (0.08 MB DOC) [file pone.0005950.s002.doc]

**Text S2**

Supporting Information to article ‘*Clinical prognostic value of RNA viral load and CD4 cell counts during untreated HIV-1 infection’*, by Korenromp-EL *et al.*

**Statistical Appendix . Univariate and multivariate regressions of relative risks of clinical HIV progression associated with higher RNA viral load and lower CD4 cell count.**

The Appendix Table shows the variation of relative risks (RR) of HIV progression due to unit differences in RNA viral load or CD4 cell count, with several possible covariates.

For RNA viral load (Table a&b), among datapoints analyzed, RRs per 10-fold higher RNA did not depend on any of the covariates tested; notably, RRs were constant over the years since seroconversion (as also shown in Figure 2a&b in the main article).

For CD4 counts (Table c&d), the RRs per 100 lower CD4 cells/uL of progression to both AIDS and death increased with the years since seroconversion (as also shown in Figure 2c&d of the main article). Time since seroconversion on its own explained 74% and 89% of variation in RRs due to CD4 cell decrease, among datapoints analyzed for progression to AIDS and death, respectively. In univariate analysis, the RR decreased with increasing median CD4 level at baseline – but this variable became non-significant in multivariate regression, due to its co-linearity with ‘years since seroconversion’. None of the other variables tested showed a significant effect on RRs.

The absence of statistical effects of duration of follow-up (see Table 1) on RRs conferred by either RNA or CD4 difference justifies our *a priori* assumption of constant hazards over the course of a typical several-year follow-up, starting from a given median timepoint since seroconversion.

**Appendix Table**. Linear regression of relative risks (RR) of HIV progression for

(a) AIDS per 10-fold higher RNA viral load; (b) death per 10-fold higher RNA viral load; (c) AIDS per 100 cells/μL lower CD4 count; (d) death per 100 cells/μL lower CD4 count.

**(a) Relative risk of AIDS per 10-fold higher RNA viral load**

| *Co-variable*  *(d.f. in univariate analysis)* | *Parameter value (significance)* | |
| --- | --- | --- |
|  | *Univariate* | *Multivariate* |
|  |  | *Model 1* |
| Years since seroconversion, at baseline CD4 measurement (15 d.f.) | 0.048 (p=.71; R^2^ =-0.056) | -0.111 (p=.73) |
| Median CD4 at baseline (14 d.f.) | -0.002 (p=.13; R^2^ =0.10) | -0.004 (p=.27) |
| Duration of follow-up (15 d.f.) | -0.070 (p=.52; R^2^ =-0.037) | 0.16 (p=.43) |
| Annual rate of progression to AIDS (15 d.f.) | -0.62 (p=.80; R^2^ =-0.062) | 4.59 (p=.55) |
| Geographical region (15 d.f.) | 1.10 (p= .11; R^2^ =0.102) | 1.89 (p= .21) |
| Use vs. non-use of antiretroviral mono-/bi-therapy: at baseline (15 d.f.) | -6.50 (p= .56; R^2^ =-0.041) | -4.67 (p= .65) |
| Use vs. non-use of antiretroviral mono-/bi-therapy: during follow-up (15 d.f.) | -0.49 (p= .71; R^2^ =-0.056) | 0.10 (p= .97) |
| Goodness of fit of multivariate model: R^2^ |  | 0.132 |

**(b) Relative risk of death per 10-fold higher RNA viral load**

| *Co-variable*  *(d.f. in univariate analysis)* | *Parameter value (significance)* | |
| --- | --- | --- |
|  | *Univariate* | *Multivariate* |
|  |  | *Model 1* |
| Years since seroconversion, at baseline CD4 measurement (8 d.f.) | 0.095 (p=.49; R^2^ =-0.054) | 0.437 (p=.50) |
| Median CD4 at baseline (5 d.f.) | -0.004 (p=.22; R^2^ =0.13) | Excluded due to collinearity |
| Duration of follow-up (8 d.f.) | -0.144 (p=.27; R^2^ =0.044) | -0.224 (p=.68) |
| Annual rate of progression to death (7 d.f.) | 1.71 (p=.77; R^2^ =-0.13) | -13.4 (p=.55) |
| Geographical region (8 d.f.) | 0.32 (p= .57; R^2^ =-0.077) | -2.59 (p=.54) |
| Use vs. non-use of antiretroviral mono-/bi-therapy: at baseline (8 d.f.) | 0.166 (p= .90; R^2^ =-0.123) | -18.72 (p=.52) |
| Use vs. non-use of antiretroviral mono-/bi-therapy: during follow-up (8 d.f.) | -1.76 (p= .41; R^2^ =-0.028) | -9.12 (p=.59) |
| Goodness of fit of multivariate model: R^2^ |  | n/a |

**c) Relative risk (minus 1)* of AIDS per 100 cells/**μ**L lower CD4 count**

| *Co-variable*  *(d.f. in univariate analysis)* | *Parameter value (significance)* | | | |
| --- | --- | --- | --- | --- |
|  | *Univariate* | *Multivariate* | | |
|  |  | *Model 1* | *Model 2* | *Model 3* |
| Years since seroconversion, at baseline CD4 measurement (14 d.f.) | **0.37 (p=<.001; R^2^ =0.74)*** | 0.34 (p=.15) | 0.29 (p=.17) | **0.41 (p=.004)** |
| Median CD4 at baseline (12 d.f.) | **-0.005 (p=.008; R^2^ =0.42)** | -0.001 (p=.66) | -0.02 (p=.61) |  |
| Duration of follow-up (13 d.f.) | -0.16 (p=.30; R^2^ =0.12) | 0.087 (p=.54) |  | 0.088 (p=.49) |
| Annual rate of progression to AIDS (12 d.f.) | 3.8 (p=.39; R^2^ =-0.016) |  |  |  |
| Geographical region (12 d.f.) | 3.8 (p=.39; R^2^ = -0.016) |  |  |  |
| Use vs. non-use of antiretroviral mono-/bi-therapy: at baseline (12 d.f.) | -2.24 (p=.90; R^2^ = -0.082) |  |  |  |
| Use vs. non-use of antiretroviral mono-/bi-therapy: during follow-up (12 d.f.) | -0.93 (p=.41; R^2^ = -0.020) |  |  |  |
| Goodness of fit of multivariate model: R^2^ |  | 0.44 | 0.48 | 0.47 |

**(d) Relative risk of death (minus 1)* per 100 cells/**μ**L lower CD4 count**

| *Co-variable*  *(d.f. in univariate analysis)* | *Parameter value (significance)* | | | |
| --- | --- | --- | --- | --- |
|  | *Univariate* | *Multivariate* | | |
|  |  | *Model 1* | *Model 2* | *Model 3* |
| Years since seroconversion, at baseline CD4 measurement (7 d.f.) | **0.30 (p<.001; R^2^ =0.89)*** | 0.29 (p=.17) | 0.35 (p=.15) | **0.26 (p=.014)** |
| Median CD4 at baseline (6 d.f.) | **-0.005 (p=.004; R^2^ =0.74)** | 0.006 (p=.27) | 0.003 (p=.58) |  |
| Duration of follow-up (6 d.f.) | -0.27 (p=.04; R^2^ =0.44) | -0.144 (p=.19) |  |  |
| Annual rate of progression to death (6 d.f.) | **12.4 (p<.007; R^2^ =0.68)** | 15.3 (p=.155) | 8.7 (p=.37) | 4.3 (p=.36) |
| Geographical region (6 d.f.) | -0.28 (p=.71; R^2^ =-0.025) |  |  |  |
| Use vs. non-use of antiretroviral mono-/bi-therapy: at baseline (6 d.f.) | 2.34 (p=.09; R^2^ =-0.305) |  |  |  |
| Use vs. non-use of antiretroviral mono-/bi-therapy: during follow-up (6 d.f.) | 1.38 (p=.64; R^2^ =-0.016) |  |  |  |
| Goodness of fit of multivariate model: R^2^ |  | 0.85 | 0.79 | 0.81 |

Bold font indicates statistical significance (p-value <0.025).

R^2^ = Proportion of variation in relative risk explained by this variable; d.f. = degrees of freedom.

* Unlike for other regressions, for the univariate regressions of ‘Relative risk of AIDS or death per 100 cells/uL CD4 decrease’ on co-variable ‘Years since seroconversion’ no constant c.q. intercept was allowed, in order to enforce a RR of 1.0 at time = 0 years since seroconversion (when CD4 is still largely defined by its pre-infection level). This restriction was implemented by applying all regressions under (c ) and (d) above on a transformation of RR into (RR-1).

Units and categories of co-variables: CD4: cells/μL; duration of follow-up: years; geographical region: USA and Western Europe versus Africa; use of ART mono- or bi-therapy at baseline: % of participants receiving ART at time of baseline RNA or CD4 measurement; use of ART mono- or bi-therapy during follow-up: (estimated) % of follow-up time with ART mono- or bi-therapy.
